# Society Meetings

**Published:** 1916-12

**Authors:** 


### SOCIETY MEETINGS

Brief reports and announcements of meetings of societies of Western New York. and those of general scope, are requested from Secretaries. Copy should be on hand the fifteenth of the month. Full transactions will be published at cost of composition.

**DOCTOR: Is your Society properly represented here? If not, please bring our standing announcement to the attention of the members.**

**The American College of Surgeons** held its annual meeting in Philadelphia, Oct. 27. Dr. George W. Crile of Cleveland was elected president, succeeding Dr. J. M. T. Finney who has served since the beginning of the organization. Drs. Rudolph Matas and Robert G. Le Conte were elected vice-presidents. 200 fellows have been admitted to the College during the past year, out of 2500 applicants, the total membership being now 3700. One of the prime requirements for fellowship is the submittance of case histories and only about 40% of those complying with this requirement were admitted because, in the opinion of the State and Central Credentials Committees, the histories were incomplete, the diagnosis not established or the surgical judgment poor. Such rejection is not necessarily permanent. One of the main functions of the College is the control of hospital management, both to secure proper surgery for the people and suitable education of surgeons during their interneship. There is mentioned with approval, a hospital which maintains the open staff policy but in which any operator not satisfying the superintendent as to his competency, is requested not to bring further patients to the hospital. The matter of fee-splitting is summed up in the words: "Anyone who divides fees is a liar and a thief." Seven charges of fee-splitting were investigated, one fellow expelled and two suspended for further evidence. Standards of moral and scientific excellence are to be established by a committee, to be elected by states, on the basis of 2 to each million of population. (Note: It seems to us that the surprising success of this organization in the brief period of its existence, is the best answer to the objections offered. Yet the authoritative way in which what is really a private institution plans its campaign and the drastic measures taken to remedy evils, are not without certain dangers. For example, the literal division of a fee, as openly and honestly practiced with the lump sum voted by congress for the professional attendance on the late President McKinley, does not warrant us in considering the honorable and eminent gentlemen involved, to be "thieves and liars." The provisions of the College in this regard, have been condemned, not from any sympathy with ordinary, sneaking fee-splitting but on the ground that they discriminate in various practical ways in favor of the surgeon and the consultant).

---

**Elmira Academy of Medicine.** The regular meeting was held Nov. 15. Dr. Chas. G. Wagner, Supt. of the Binghamton State Hospital gave a paper on Mental Hygiene and Dr. Karl Connell showed lantern slides illustrating experience "With the Troops at the Mexican Border, and Sanitary Service in France and the Central Empires." A collation was served. The President is Dr. La Rue Colegrove; the Secretary Dr. E. T. Bush.

---

**Rochester Academy of Medicine.**. A regular meeting of Section III was held Nov. 8. Drs. Norris G. Orchard, Franklin W. Bock and Charles R. Witherspoon, spoke, respectively, on Mental Hygiene, "Guarding the Portals of Entry" and Diet, of the First Two Years of Childhood. Dr. Lucius L. Button, Chairman; Dr. W. Douglas Ward, Secretary.

---

**The Monroe County and Western N. Y. Homeopathic Medical Societies** held a joint meeting Nov. 14, under the presidency of Dr. R. Montfort Schley of the latter and Dr. Wm. Perrin of the former, Dr. Lloyd H. Clark, secretary. The scientific program was as follows:

Pathology of Gall-Bladder and Bile-Ducts, Dr. Warren C. Daly, discussion opened by Dr. H. R. Brown, Pathologist Rochester Homeopathic Hospital; Diagnosis, Dr. George R. Critchlow, discussion opened by Dr. Davis B. Jewett; Roentgen Rays in Diagnosis, Dr. Llewellyn J. Sanders, discussion opened by Dr. Edgar B. Cook; Surgery of Gall-Bladder and Bile-Ducts, Dr. Frank T. Bascom, discussion opened by Dr. Hiland G. Shepard; Homeopathic Therapeutics, Dr. Volney A. Hoard, discussion opened by Dr. Charles R. Sumner.

Dinner was served to the members and ladies and in the evening, Dr. Edmund Boddy gave a talk on Medical Relief Work in the Balkans.

---

**U. of B. District Branch Alumni Meetings.** Chautauqua District Alumni Association, Thursday, Nov. 16, 1916. Holley House, Bradford, Pa. Central and Northern New York Alumni Association, Thursday, Apr. 5, 1917, Syracuse, N. Y. Third Annual Dinner, Federated Alumni, Thursday, Feb. 22, 1917, Buffalo, N. Y. Interstate Alumni Association, Monday, Mar. 26, 1917, Binghamton, N. Y. Metropolitan Alumni Association, Tuesday, Mar. 27, 1917, New York City. Rochester

District Alumni Association, Thursday, Apr. 19, 1917, Rochester, N. Y.

---

At the dinner of the **Chautauqua District Assn.**, held at the Holley Hotel, Bradford, Pa., Nov. 16, the President, Dr. J. E. K. Morris of Olean, acted as toastmaster. Mayor Herman H. North gave an address of welcome and speeches were made by Drs. Richard F. Morgan, Pres. of the Federated Alumni; T. H. McKee, Dean of the Medical Dept.; L. Kauffman, ex-Pres. of the Medical Alumni; Willis G. Gregory, Dean of the Pharmacy Dept.; D. H. Squire, Dean of the Dental Dept.; W. D. Greene, Pres. of the Medical Alumni. The following officers were elected for the Chautauqua Branch, for the next year: Pres., Dr. H. J. Nichols, Bradford; Vice-Pres., John C. Knight, Salamanca; John J. Mahoney, Jamestown, and E. L. Todd, Dunkirk; Sec., J. D. Earty, Olean; Treas., Geo. W. Ross, Wellsville. The fourth annual meeting will be held at Salamanca, Nov., 1917.

---

**Buffalo Academy of Medicine**, Section of Pathology, Oct. 25.

Rabies as a Public Health Problem, Miss Ethel A. Jacobs, Prof. of Bacteriology, University of Buffalo, discussion by Drs. Carpenter, Thibaudeau and Dr. Ell. Volgenau, Veterinary City Health Department; Some Aspects of Hodgkins Disease, J. A. P. Millet, M. D., Internist at Hospital, State Institute for the Study of Malignant Disease, Lantern Slide Demonstration, discussion by Drs. Harvey R. Gaylord, Herbert U. Williams. Wm. F. Jacobs, M. D., Chairman; Wm. G. Bissell, Secretary.

Section of Surgery, Nov. 1. Clinical Data with Nephrolithasis, Lantern Slide Demonstration, Dr. Wm. F. Braash, Chief of Urological Department, The Mayo Clinic, Rochester, Minn., discussion opened by Dr. J. A. Gardner. F. M. O'Gorman, M. D., Chairman; J. B. Cross, M. D., Secretary.

Section of Medicine, Nov. 8. Some Subjects of Psychoanalysis, Smith Ely Jeliffe, M. D., Ph. D., Adjunct Professor of Diseases of the Mind and Nervous System in N .Y. Post Graduate Medical School and Hospital. Edward A. Sharp, M. D., Chairman; Harry Mead, M. D., Secretary.

Section of Obstetrics and Gynecology, Nov. 15. The Interrelationship of the Pituitary and Female Sex Glands and its Clinical Application, James E. King, M. D., the paper was illustrated by lantern slides. Irving W. Potter, M. D., Chairman; Herman K. DeGroat, M. D., Secretary.

Section of Pathology, Nov. 22. "Some of the Practical Problems in Bacteriology and Pathology Encountered in the War Zone," Lantern Slide Demonstration, John J. Mackenzie, Prof. of Path. and Bact., Univ. of Toronto. Dr. Wm. F. Jacobs, Chairman; Dr. Wm. G. Bissell, Secretary.

